# Laparoscopic Repair of Small Bowel Obstruction Caused by an Intersigmoid Hernia: A Case Report

**DOI:** 10.7759/cureus.36793

**Published:** 2023-03-28

**Authors:** Esere A Nesiama, Lajohn Quigley, Haris Nazim, Sameer Prakash, Izi Obokhare

**Affiliations:** 1 General Surgery, Texas Tech University Health Sciences Center, Lubbock, USA; 2 General Surgery, Texas Tech University Health Sciences Center, Amarillo, USA; 3 Internal Medicine, Memorial Hermann The Woodlands Hospital, Spring, USA

**Keywords:** enema, intersigmoid fossa, laparoscopic hernia repair, lower gi or colorectal surgery, laparoscopic colorectal surgery

## Abstract

Bowel obstructions can be caused by internal hernias which are protrusions of the bowel into openings within the abdominal cavity. There are various types of internal hernias including sigmoid hernias which involve the sigmoid mesentery.Sigmoid hernias are very difficult to diagnose clinically, even with the aid of radiologic imaging. Computed tomography (CT) scan findings often reveal small bowel obstructions; however, they are not sensitive to intersigmoid hernias. Most of these rare herniations are repaired by open abdominal surgery followed by the closure of the mesenteric defect to prevent a recurrence. We present the case of a 57-year-old man who presented to the emergency department with a small bowel obstruction that was caused by an intersigmoid hernia and was successfully repaired through a minimally invasive laparoscopic approach. This case demonstrates an intra-operative diagnosis of an intersigmoid hernia and reviews the benefits of a laparoscopic approach for the reduction of the sigmoid mesentery.

## Introduction

Internal hernias are defined as protrusions of the bowel into openings within the abdominal cavity and cause 0.5%-6% of all cases of bowel obstruction [1,2]. While there are various types of internal hernias, sigmoid hernias involve the sigmoid mesentery and account for 6% of all documented internal hernias [2-4]. Furthermore, sigmoid hernias can be classified into three distinct categories based on their characteristics: intersigmoid, transmesosigmoid, and intramesosigmoid. The determining characteristics for classification are a result of sigmoid mesocolon embryologic development which influences the anatomical variations in hernial presentation. The sigmoid mesocolon is a fold of the peritoneum that typically attaches the sigmoid colon to the pelvic wall [2]. During normal embryologic development, the left leaf of the sigmoid mesentery fuses to the parietal peritoneum at around five months of gestation [5]. The folding and incomplete fusion of the mesentery sometimes creates an inverted V-shaped opening in the sigmoid mesentery termed the intersigmoid fossa [6]. While this opening is considered a defect in the mesentery, it is estimated to occur in 50%-75% of individuals based on autopsy reports [5,6]. The orifice typically presents on the left inferior aspect which is why it can most easily be located by turning the sigmoid colon superiorly and to the right [7].

Sigmoid hernia cases are very difficult to diagnose clinically because radiologic imaging is sensitive for the hernias but the imaging lacks specificity for cases that are not partial obstructions and require surgical management [8]. However, with prompt surgery, sigmoid hernias are often easily diagnosed, reducible, and associated with an excellent prognosis. Surgical treatment for small bowel obstruction consists of two main approaches with varying benefits depending on the situation: open laparotomy and minimally invasive laparoscopy. In this case, a small bowel obstruction caused by an intersigmoid hernia was successfully repaired through a laparoscopic approach. This article was previously presented as a meeting abstract poster at the 2023 North and South Texas Chapters ACS Joint Annual Meeting on February 23, 2023.

## Case presentation

**Figure 1:**
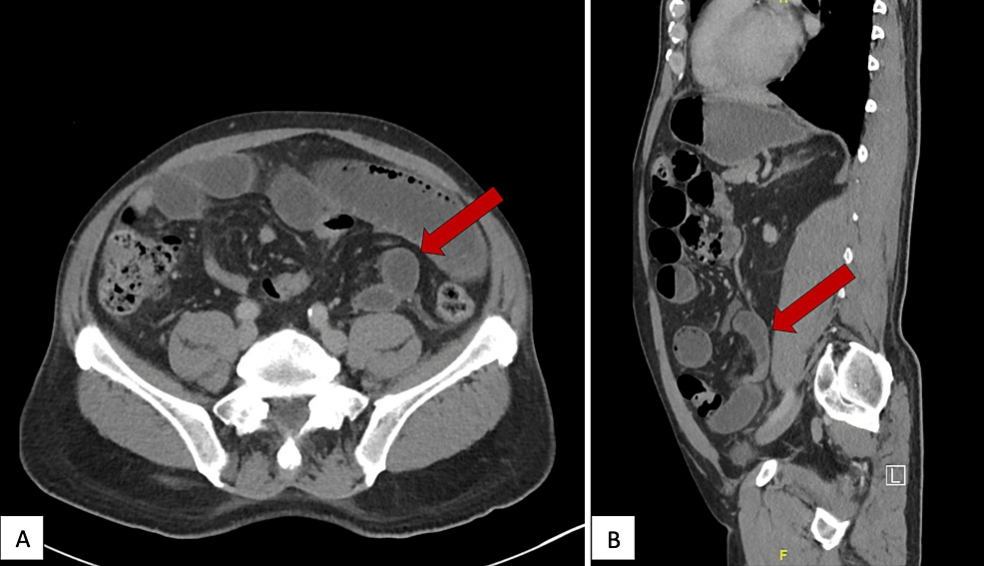
Preoperative contrast-enhanced CT taken upon admission suggested a complete or high-grade small bowel obstruction. 1A: Transverse contrast-enhanced CT with an arrow delineating encapsulated fluid-filled loops of bowel in the left lower abdomen near the sigmoid mesocolon. 1B: Sagittal contrast-enhanced CT with an arrow delineating obstruction in the left hemipelvis with proximal dilation of the small bowel.

**Figure 2:**
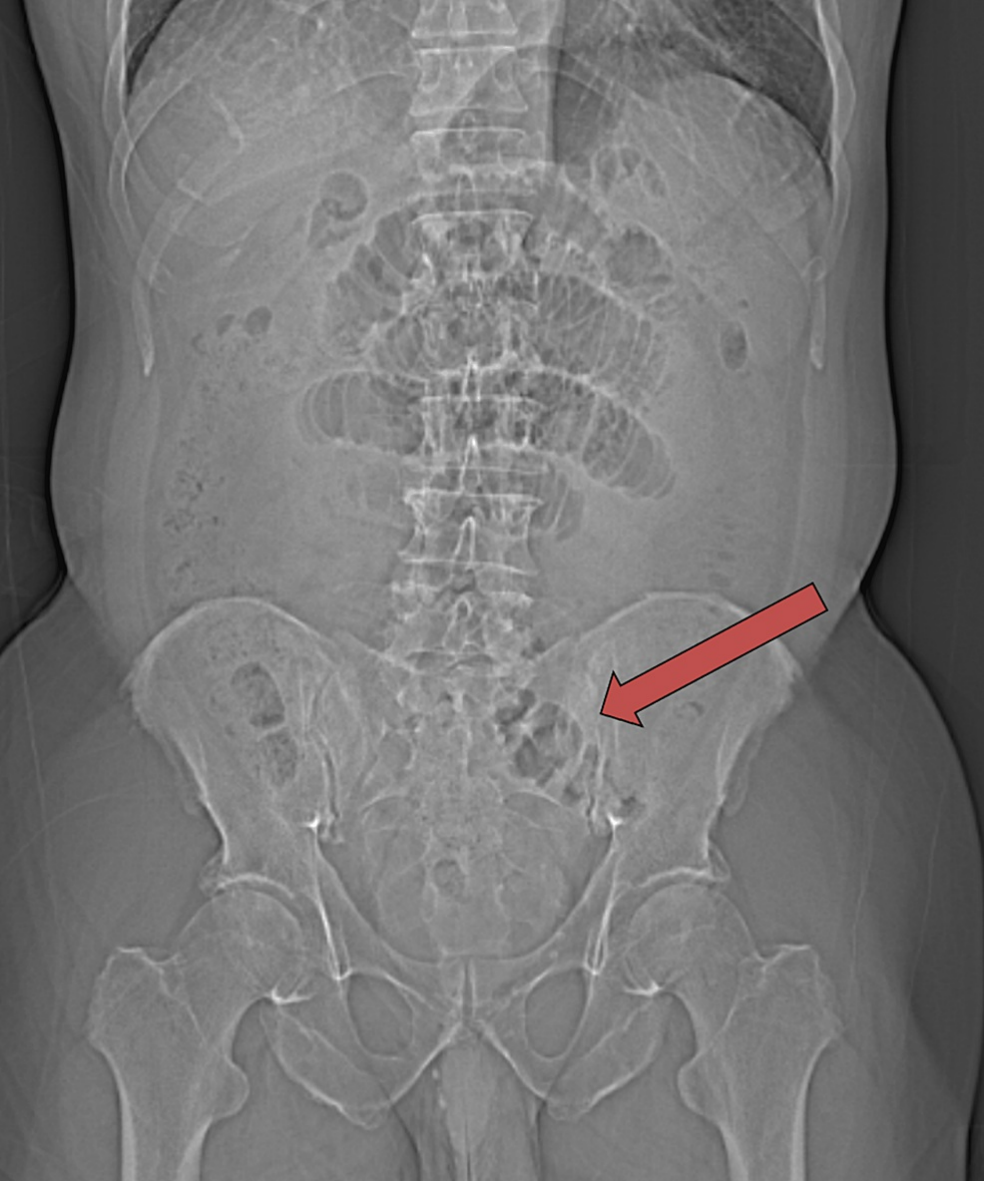
Anterior-posterior X-ray of the abdomen with arrow pointing to one of the many air-fluid levels found preoperatively.

A 57-year-old male presented to the emergency department complaining of abdominal symptoms that included nausea, emesis, diffuse constant lower abdominal pain, and obstipation. The symptoms began shortly after he strained to start a boat motor three days ago. On initial presentation, he reported 10 episodes of emesis in the last 24 hours and no flatus or bowel movements for the past three days. His past medical history included a diagnosis of schizophrenia, however, he denied having any past surgical history or previous abdominal surgeries. Complete blood count, comprehensive metabolic panel, and electrolytes on admission were within normal limits except for noted hypokalemia of 3.5 mmol/L which was subsequently corrected with fluid resuscitation. CT scan of the abdomen and pelvis with contrast demonstrated a distended stomach, air-fluid levels, and distention of the small bowel down to the region of the left hemipelvis with completely decompressed loops of the distal small bowel (Figures 1-2). The radiology report suggested a likely complete or high-grade small bowel obstruction from a hernia.

A nasogastric tube was initially placed to decompress the stomach. Due to the duration and severity of the symptoms combined with a lack of surgical history, the patient was promptly taken to the operating room for a diagnostic laparoscopy. During the procedure, distal decompression of the small bowel and dilation of the proximal small bowel was noted. A defect was visualized in the sigmoid mesentery with a loop of small bowel protruding through it. A transition point was also found within this defect of the sigmoid mesentery. The small bowel was surgically reduced, watched, and noted to have a healthy color with visible peristalsis post-reduction. No bowel resection was required during the procedure and the mesenteric defect was closed by a 3-0 Ethicon polydioxanone (PDS) (Ethicon Inc, Somerville, NJ) interrupted suture to prevent a recurrence. The patient had a normal recovery without complications and was discharged two days following the operation. In his two-week post-operative follow-up, there were no complaints on concerns indicative of further interventions.

## Discussion

Internal hernias are rare causes of bowel obstruction, and they are often difficult to diagnose preoperatively. Sigmoid hernias, which involve intestinal herniation through the sigmoid mesentery, account for up to 6% of all internal hernias and are responsible for roughly one out of every 800 cases of small bowel obstruction [2-4]. The relative frequency of hernia types is outlined in Table 1 with different sigmoid hernia frequencies outlined in Table 2.

**Table 1:** Relative frequency of sigmoid hernias compared to other internal hernias. [2-4]

Type of Hernia	Relative Frequency (%)
Sigmoid	6
Foramen of Winslow	53
Paraduodenal	8
Transmesenteric	1-4
Transomental	13
Pericecal	6
Supravesical and pelvic	6

**Table 2:** Relative frequency of the different classification categories of sigmoid hernias. [5]

Type of Sigmoid Hernia	Relative Frequency (%)
Intersigmoid	24.5-35
Intramesosigmoid	50-57.3
Transmesosigmoid	15-18

Intestinal protrusions through openings in this mesentery, whether acquired or congenital, form an internal sigmoid hernia. Intersigmoid hernias are typically divided into three categories based on their characteristics: intersigmoid hernias, transmesosigmoid hernias, and intramesosigmoid. Intersigmoid hernias involve the incarceration of the bowel through the intersigmoid fossa [4-7,9-10]. Transmesosigmoid hernias occur when the intestine herniates through a defect in both the left and right leaves of the sigmoid mesentery. Finally, intramesosigmoid hernias occur when the bowel herniates through either the left or the right leaf of the mesocolon [9]. The majority of internal hernias involving the sigmoid mesentery are intersigmoid hernias, however, transmesosigmoid hernias have the greatest rates of bowel resection and mortality [5,9,11].

Since the intersigmoid fossa is a relatively common anatomical finding, there is a lot of speculation surrounding risk factors for its development. Some literature suggests that the entrapment of the small intestine in the pelvic cavity by adhesions secondary to previous abdominal surgeries is a risk factor for an intersigmoid hernia [6]. Some literature also suggests that previous surgeries may create or enlarge the opening of the intersigmoid fossa [6]. Despite these suggested risk factors, the majority of patients found to have intersigmoid hernias, such as our patient, have no history of previous abdominal surgeries [9]. As with other types of internal hernias, excessive length of the small intestine mesentery and increased mobility of the intestines may predispose individuals to develop intersigmoid hernias [6]. Finally, some literature asserts that the intersigmoid fossa becomes smaller over time, and therefore the risk of developing an intersigmoid hernia is inversely associated with age [7,12].

Due to the rarity and lack of clear clinical or radiological features, intersigmoid hernias are often found, as in our case, during surgical exploration of the abdomen indicated for an acute small bowel obstruction [3-13]. CT scans may demonstrate sacculated loops of the intestine that occupy the left lower quadrant in combination with a medial displacement of the sigmoid colon [14]. CT scans may otherwise show a cluster of Y and X-shaped dilated loops of bowel trapped posterior to the sigmoid colon [14]. While the CT findings often reveal small bowel obstruction, they are not sensitive to intersigmoid hernias. Barium or gastrograffin enemas are described as effective means of diagnosis with indocyanine green angiography use as a consideration for intraoperative visualization based on physician preference [8,10]. They are documented in the literature to allow visualization of small bowel loops encapsulated between loops of the sigmoid colon [8,10]. Increased awareness of intersigmoid hernias and the use of enemas may lead to changes in the frequency and accuracy of their pre-operative diagnosis.

While the first successful surgical repair of intersigmoid hernias was described in 1910, there is currently still no agreed-upon standard method of repair [13]. Current literature discusses the variety of available surgical techniques used to treat intersigmoid hernias. The majority of these cases have historically undergone open abdominal surgery for diagnostic and treatment purposes [9]. Recent reports, including our case, demonstrate the efficacy of a laparoscopic approach [5,10,15]. The advantages of laparoscopy over laparotomy include shortened hospital stay, shorter recovery, decreased pain, decreased risk of infection, and fewer complications. Some literature asserts that laparoscopy does not allow adequate space for bowel resection and anastomosis if necessary, however, the large majority of intersigmoid hernias (85%) do not require any bowel resection at all [9]. 

Debates exist surrounding the necessity to close the intersigmoid fossa after the reduction of the herniated intestine. The majority of surgeons advocate for the closure of the intersigmoid fossa in order to prevent the recurrence of herniation through the opening [5,6,9,15]. However, one article claims that recurrence is rare, and incarceration of the bowel is even less likely for larger orifices [10]. Therefore, they suggest that intersigmoid fossas do not require closure and instead describe enlarging the opening in the mesentery as an effective means of preventing bowel incarceration [10]. We support the use of a suture to close the opening in the sigmoid mesentery to prevent the recurrence of this internal hernia.

## Conclusions

Our case demonstrates an intra-operative diagnosis of intersigmoid hernia and demonstrates the effectiveness of using a laparoscopic approach to reduce the herniated intestine to close the opening in the sigmoid mesentery. While there is insufficient evidence to verify superiority, we recommend a laparoscopic approach for intersigmoid hernia treatment as well as the closure of the mesenteric defect to prevent a recurrence.
